# Total nephrectomy following *Corynebacterium coyleae* urinary tract infection

**DOI:** 10.1099/jmmcr.0.005149

**Published:** 2018-04-06

**Authors:** Claudia M. Barberis, Eduardo Montalvo, Soledad Imas, Germán Traglia, Marisa N. Almuzara, Carlos Hernán Rodriguez, Angela Famiglietti, Octavio Mazzocchi, Carlos Vay

**Affiliations:** ^1^​ Universidad de Buenos Aires. Facultad de Farmacia y Bioquímica, Departamento de Bioquímica Clínica, Cátedra de Microbiología Clínica, Hospital de Clínicas José de San Martín, Ciudad Autónoma de Buenos Aires, Argentina; ^2^​ Universidad de Buenos Aires. Departamento de Medicina, V Cátedra de Medicina Interna, Hospital de Clínicas José de San Martín, Ciudad Autónoma de Buenos Aires, Argentina

**Keywords:** *Corynebacterium coyleae*, urinary tract infection, nephrectomy

## Abstract

**Introduction:**

*Corynebacterium coyleae* is a Gram-stain-positive non-lipophilic coryneform rod first described in blood samples and pleural fluid. There is scarce information about the clinical relevance of *C. coyleae* and none on complicated urinary tract infections has been described so far.

**Case presentation:**

A 36-year-old woman with a history of chronic kidney failure, under thrice-weekly haemodialysis since 2014 due to polycystic kidney disease, presented with hypogastric pain, lower left quadrant pain and nausea. Since 1997, the patient had developed several episodes of urinary tract infection. On admission, the patient presented tenderness in the lower abdomen and fist positive lumbar percussion. Urine culture showed significant bacterial growth (>10^5^ c.f.u. ml^−1^). Slightly glistening colonies of 1 mm in diameter were observed after a 24 h incubation. Gram staining showed coryneform Gram-stain-positive rods. The patient was diagnosed as having a complicated urinary tract infection. A bilateral nephrectomy was performed on the fourth day of hospitalization. Two samples of kidney tissue were sent for culture. Direct examination of the material revealed the presence of abundant inflammatory reaction and Gram-positive diphtheroid rods. The organism was identified using MALDI–TOF and conventional biochemical tests; in both isolates further identification was performed by PCR amplification and sequence analysis of the *rpoB* gene as *Corynebacterium coyleae*.

**Conclusions:**

*C. coyleae* is an infrequent species among the genus *Corynebacterium* that should be considered as an emerging pathogen that can be involved in nosocomial infections and complicated urinary tract infections

## Introduction

*Corynebacterium coyleae* is a Gram-stain-positive non-lipophilic coryneform rod first described by Funke *et al*. in blood samples and pleural fluid [1].

There is scarce information about the clinical relevance of *C. coyleae* and none on complicated urinary tract infections has been described so far, to our knowledge [2]. *C. coyleae* appears to be an emerging pathogen and its clinical significance should be considered in urine, in addition to other specimens, especially in nosocomial infections and immunosuppressed patients [1, 2].

## Case report

A 36-year-old woman with a history of chronic kidney failure, under thrice-weekly haemodialysis since 2014 due to polycystic kidney disease, presented with hypogastric pain, left lower quadrant pain and nausea. Since 1997, the patient had developed several episodes of urinary tract infection (UTI). The last UTI occurred one month before hospitalization, and she received ciprofloxacin treatment. On admission, she also presented tenderness in the lower abdomen and fist positive lumbar percussion. Laboratory testing revealed 9800 white blood cells mm^−3^ and elevated urea and creatinine levels (158 mg dl^−1^ and 10.2 mg dl^−1^, respectively).

Kidney ultrasound revealed a bilateral 30 mm pyelocalyceal dilatation and multiple cysts in both kidneys.

The midstream urine sample was subject to microbiological testing. The urine testing showed 15–20 leukocytes per high-power field (HPF) and 2–5 erythrocyte per HPF. The urine sample was aerobically cultured on CLDE agar (Britania) and on chromogenic agar CPS ID medium (bioMérieux) at 35 °C. Urine culture showed significant bacterial growth (>10^5^ c.f.u. ml^−1^). Slightly glistening colonies of 1 mm in diameter were observed after a 24 h incubation. Gram staining showed coryneform Gram-positive rods.

The isolate was identified by using conventional biochemical tests according to the identification scheme proposed by Funke *et al*. and adapted by Bernard [1, 3]. Colonies were about 1–1.5 mm in diameter after 24 h (non-lipophilic colonies). Catalase reaction was positive, fermentative metabolism was observed as well as a strong positive CAMP reaction. Pyrazinamidase and alkaline phosphatase were also positive. Nitrate reduction, β-glucuronidase, β-galactosidase, α-glucosidase, *N*-acetyl-β-glucosaminidase, aesculin, gelatin hydrolysis and urease were all negative. Acid was only produced from glucose.

Mass spectra were acquired using a MALDI–TOF MS spectrometer in a linear positive mode (Microflex, Bruker Daltonics) and were analyzed in an m/z range of 2.000 to 20.000. The MALDI Biotyper library version 3.0 and MALDI Biotyper software version 3.1 were used for bacterial identification. The MALDI–TOF MS analysis showed a score of 1.979 for *Corynebacterium coyleae*.

Antimicrobial susceptibility was determined using the E-test technique (bioMérieux) on Mueller–Hinton agar supplemented with 5 % sheep blood and the inoculum size was equivalent to a No. 0.5 Mc Farland standard [4]. Plates were incubated aerobically at 37 °C for 24 h. Minimum inhibitory concentration (MIC) results showed resistance to penicillin (8 µg ml^−1^), ceftriaxone (32 µg ml^−1^), trimethoprim-sulfamethoxazole (TMS) (64 µg ml^−1^), susceptibility to vancomycin (0.5 µg ml^−1^), and intermediate susceptibility to ciprofloxacin (2 µg ml^−1^). The interpretative categories for the MICs obtained were used following Clinical and Laboratory Standards Institute (CLSI), M45 [5].

The patient was diagnosed as having a complicated urinary tract infection. She was empirically treated with piperacillin–tazobactam at 2.25 g every 12 h and 750 mg post-haemodialysis and then rotated to ciprofloxacin 400 mg every 12 h for 14 days. Due to her clinical condition (chronic kidney failure, polycystic kidneys) and several recurrent urinary tract infections, a bilateral nephrectomy was performed on the fourth hospitalization day. A supraumbilical laparotomy was carried out and a splenectomy was performed due to surgical complications.

**Fig. 1.**
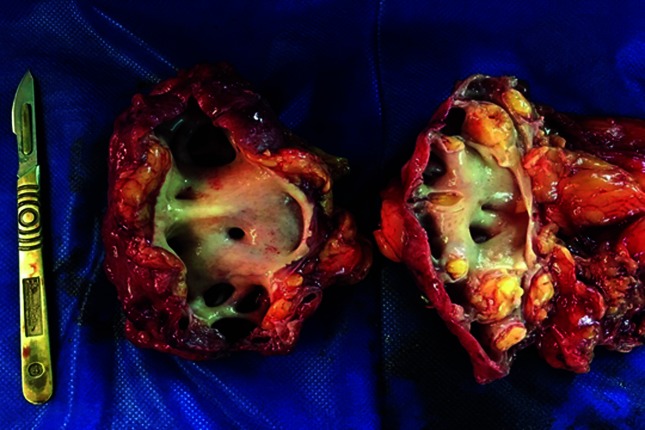
Morphological characteristics of both kidneys with thinning of the kidney parenchyma and medullary–cortical alteration with pyelocalyceal dilatation.

Two samples of kidney tissue were sent for culture (Fig. 1).

Direct examination of the material revealed the presence of abundant inflammatory reaction and Gram-stain-positive diphtheroid rods.

Since no microbial growth was observed at 24 h incubation, neither in 5 % sheep blood agar nor in chocolate agar incubated in 5 % CO_2_, we inoculated a bottle of Bactec Aerobic/F culture medium in order to neutralize the antibiotic effect from both samples. Culture plates were incubated for several days, however, at 24 h a positive culture was obtained from Bactec Aerobic/F culture medium. The organism was identified using MALDI–TOF and conventional biochemical tests, and in both isolates further identification was performed by PCR amplification and sequence analysis of the *rpo*B gene. The PCR product of the *rpo*B gene, using the primers described by Khamis *et al*. [6, 7] was generated with Taq DNA polymerase based on the manufacturer’s specifications (Qiagen). Sequencing of the 450 bp PCR product was performed on both DNA strands using an ABI Prism 3100 BioAnalyzer equipment at the Macrogen Inc. sequencing facility, Seoul, Republic of Korea. The sequences were analysed with the blast V2.0 software (http://www.ncbi.nlm.nih.gov/BLAST/). Sequence analysis revealed a 96 % identity with the sequences corresponding to the *rpo*B gene of *Corynebacterium coyleae.* The GenBank accession number for the *rpoB* gene sequence is MG764567.

**Fig. 2.**
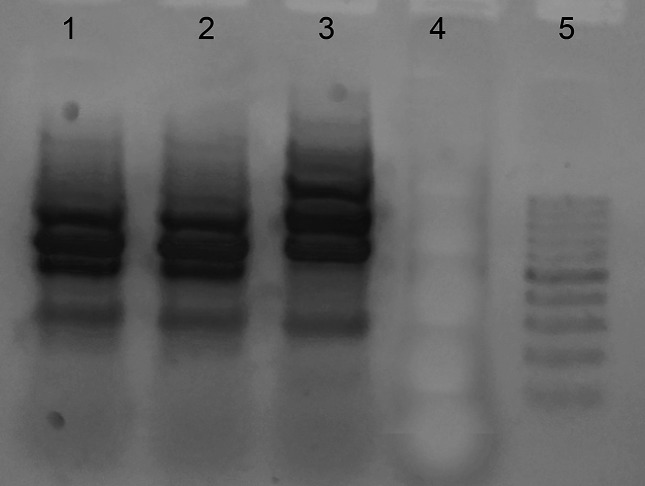
The DNA amplification patterns obtained by DO-PCR were identical in both samples (urine and kidney tissue). Line 1 *C. coyleae* from urine sample, line 2 *C. coyleae* from kidney tissue, line 3 *C. coyleae* strain from collection.

To determine the relatedness of the two strains, a PCR assay using degenerate oligonucleotide primers (DO-PCR) [8] was carried out. The DNA amplification pattern obtained by DO-PCR was identical in both samples (urine and kidney tissue; Fig. 2).

## Discussion

In recent years, there has been an interest in rare or infrequently described species such as members of the genus *Corynebacterium*, which are emerging opportunistic pathogens.

In relation to *Corynebacterium coyleae*, scarce published data is available from clinically significant samples. In the original description *C. coyleae* strains were derived from sterile body fluids including blood pleural fluid [1]. A few years later, Bernard *et al*. reported the isolation of eight *C. coyleae* from blood, one from abscess and the first recovery of this species in three urine samples, although not enough information on their clinical relevance was presented [9]. Then, Fernandez Natal *et al*. reported 14 *C. coyleae* isolates recovered from clinical samples from 12 in-patients, only six were considered clinically significant (bacteraemia, sepsis and soft tissue infection) but no urinary tract infection was described [2].

The isolation of a pure culture of coryneform bacteria indicates a definite criterion in terms of clinical significance. In our description not only was *Corynebacterium coyleae* isolated as a unique strain growing in a urine sample but also in both kidney tissue samples obtained intra-surgically. It is clear that repeated isolation of a pure culture of coryneform bacteria indicates an aetiological relationship to the patient’s disease.

In relation to phenotypic identification, *C. coyleae* should be differentiated from other corynebacteria such as *Corynebacterium afermentans* ss *afermentans* and *Corynebacterium auris,* which are non-lipophilic and show a positive CAMP test. Although *C. coyleae* has a fermentative metabolism, the slow fermentative acid production from glucose demands the differentiation between these oxidative corynebacteria, and also from some related genera, such as *Turicella*. In our case, differentiation from members of the genus *Turicella* was achieved by Gram morphology and the absence of DNA hydrolysis. Acid production from glucose and a positive PYR reaction (pyrrolidonilarilamidase production) allowed us to differentiate *C. auris* from *C. afermentans*.

In recent decades, the introduction of MALDI–TOF into routine diagnosis has become an irreplaceable tool in the clinical microbiology laboratory since it significantly speeds up species identification when compared with phenotypic identification. Moreover, the accuracy of MALDI–TOF as an identification method in Gram-stain-positive rods has been described by our study group and a lower cut-off score (≥1.7) has been proposed as suitable to identify Gram-stain-positive rods at the species level; also equivalent, in test accuracy, to the cut-off recommended by the manufacturer (≥2.0) [10].

Regarding molecular identification, since 16 S RNA divergence value between *C. coyleae* and *C. afermentans* is very close (<2 %) to allow precise identification [1], both isolates were definitely identified by using the sequencing of the small fragment of the *rpo*B gene. Khamis *et al*. proposed that two *Corynebacterium* isolates represented members of the same species if they showed 95.0 % or greater similarity [7], therefore *rpo*B is a simpler and more efficient tool to identify coryneform bacteria that belong to genomically different species but exhibit higher levels of 16S rRNA relatedness.

The patient was treated empirically with piperacillin–tazobactam, however, our isolate was resistant to β-lactam antibiotics (penicillin and ceftriaxone).

In recent years, there has been an increase in β-lactam antibiotic resistance in species of the genus *Corynebacterium*, particularly in *Corynebacterium striatum*, *Corynebacterium amycolatum*, *Corynebacterium urealyticum, Corynebacterium jeikeium* and *Corynebacterium riegelii*, among others. The mechanism of resistance to β-lactam antibiotics in bacteria of the genus *Corynebacterium* is still unknown; however, resistance is probably due to decreased membrane permeability or decreased affinity for these antibiotics [11]. Resistance to β-lactam antibiotics is not usually described in *C. coyleae*, however the emergence of multi-resistant strains in species of the genus *Corynebacterium* could be related to hospitalization and previous antibiotic use. These are risk factors which could play a role in the appearance of resistance in these microorganisms. Isolates from hospitalized patients could show significantly higher resistance to antibiotics than those from outpatients [12].

Our isolate showed an intermediate level of susceptibility to ciprofloxacin, however, the outcome of the patient was favourable. The use of fluoroquinolones for such recurrent infections raises the possibility of accelerated development of resistance [13]. Resistance to fluoroquinolones has been observed to be associated with point mutations within the structural gene region of gyrase subunit A. Mutations are spontaneous in nature, leading to changes in the amino acid sequences, on which the range of resistance to certain fluoroquinolones [11] depends. In this patient, not only could high doses of ciprofloxacin have lead to high urinary concentration and clinical cure after treatment but the surgical procedure may also have had an effect.

Species of the genus *Corynebacterium* can usually show decreased susceptibility to the antibiotics commonly used in UTIs, particularly ciprofloxacin or trimethoprim–sulfamethoxazole.

*C. coyleae* is an infrequently observed species among the members of the genus *Corynebacterium*, which should be considered as an emerging pathogen that can be involved in nosocomial infections and complicated urinary tract infections. Empirical use of quinolones is not a recommended, and the isolation of a clinically significant species of the genus *Corynebacterium* always requires the performance of antimicrobial susceptibility tests.
